# Genetic Feedback Regulation of Frontal Cortical Neuronal Ensembles Through Activity-Dependent Arc Expression and Dopaminergic Input

**DOI:** 10.3389/fncir.2016.00100

**Published:** 2016-12-06

**Authors:** Surjeet Mastwal, Vania Cao, Kuan Hong Wang

**Affiliations:** Unit on Neural Circuits and Adaptive Behaviors, Clinical and Translational Neuroscience Branch, National Institute of Mental HealthBethesda, MD, USA

**Keywords:** activity-dependent genetic feedback, neuronal ensembles, neuromodulation, dopamine, Arc/Arg3.1, frontal cortical circuits, learning, development

## Abstract

Mental functions involve coordinated activities of specific neuronal ensembles that are embedded in complex brain circuits. Aberrant neuronal ensemble dynamics is thought to form the neurobiological basis of mental disorders. A major challenge in mental health research is to identify these cellular ensembles and determine what molecular mechanisms constrain their emergence and consolidation during development and learning. Here, we provide a perspective based on recent studies that use activity-dependent gene *Arc/Arg3.1* as a cellular marker to identify neuronal ensembles and a molecular probe to modulate circuit functions. These studies have demonstrated that the transcription of Arc is activated in selective groups of frontal cortical neurons in response to specific behavioral tasks. Arc expression regulates the persistent firing of individual neurons and predicts the consolidation of neuronal ensembles during repeated learning. Therefore, the Arc pathway represents a prototypical example of activity-dependent genetic feedback regulation of neuronal ensembles. The activation of this pathway in the frontal cortex starts during early postnatal development and requires dopaminergic (DA) input. Conversely, genetic disruption of Arc leads to a hypoactive mesofrontal dopamine circuit and its related cognitive deficit. This mutual interaction suggests an auto-regulatory mechanism to amplify the impact of neuromodulators and activity-regulated genes during postnatal development. Such a mechanism may contribute to the association of mutations in dopamine and Arc pathways with neurodevelopmental psychiatric disorders. As the mesofrontal dopamine circuit shows extensive activity-dependent developmental plasticity, activity-guided modulation of DA projections or Arc ensembles during development may help to repair circuit deficits related to neuropsychiatric disorders.

## Introduction

Mental functions involve coordinated activities among specific groups of neurons, or neuronal ensembles, that are embedded in complex brain circuits (Hebb, 1949; Harris and Shepherd, 2015). The intrinsic excitability, synaptic connectivity and neuromodulatory inputs of individual neurons constrain the dynamic flow of neural activity in these ensembles (Bargmann and Marder, 2013; Buzsáki and Mizuseki, 2014; Gjorgjieva et al., 2016). Lack of normal constraints in neural dynamics is considered to form the neurobiological basis of mental disorders (Rolls et al., 2008; Akil et al., 2010; Deisseroth, 2014). The configurations of neuronal ensembles are established under genetic instruction during development and modified by postnatal experience and activity (Sur and Rubenstein, 2005; Takesian and Hensch, 2013; Josselyn et al., 2015; Tonegawa et al., 2015). Although human genetic studies of neurodevelopmental psychiatric disorders have implicated hundreds of risk genes, linking these genes and the molecular events they regulate within the cell to disorders at the behavioral level is a major challenge (Krystal and State, 2014; Mullins et al., 2016). A critical barrier arises from the difficulty of identifying specific neuronal ensembles that transduce the impact of genetic perturbations into behavioral consequences.

To identify functional neuronal ensembles embedded in complex circuits, electrophysiological approaches typically look for correlated activation that often occurs in small subsets of neurons scattered throughout the brain volume (Buzsáki and Mizuseki, 2014). Separating these particular neuronal populations for selective functional dissection and manipulation is not straightforward. Although molecular genetic studies have been able to define certain cell types by identifying their static gene expression signatures, such signatures often do not differentiate excitatory neuronal ensembles that are detected according to functional criteria (Huang, 2014; Angelakos and Abel, 2015).

To bridge the gap between traditional molecular genetic and neurophysiological approaches, neural activity-induced gene expression patterns have been used to identify functional ensembles (Guzowski et al., 2005; Barth, 2007). These inducible immediate early genes (IEGs) include both transcription factors and synaptic molecules, such as c-Fos and Arc/Arg3.1, respectively (Greenberg et al., 1986; Link et al., 1995; Lyford et al., 1995). Initially, there were concerns in this field that the induction of these genes might only reflect metabolic activation or general arousal, but not carry any stimulus-specific information at the cellular level. However, by analyzing *in situ* the subcellular localization of induced Arc mRNA over minutes (Guzowski et al., 1999) or tracking *in vivo* an Arc-promoter-driven fluorescent reporter over days (Wang et al., 2006), it became apparent that different natural stimuli induce Arc gene expression in distinct groups of neurons in the hippocampus or visual cortex, demonstrating the functional specificity of Arc-expressing neuronal ensembles.

Unlike some other IEGs that are broadly expressed in many different cell types, Arc is selectively induced in groups of telencephalic projection neurons under physiological conditions (Vazdarjanova et al., 2006). Furthermore, Arc interacts with excitatory postsynaptic receptors and adaptors, and plays a more direct role in regulating synaptic functions (Chowdhury et al., 2006; Zhang et al., 2015). Recent large scale human genetic studies have shown that disruptive mutations affecting Arc-interacting postsynaptic complex are selectively enriched in neurodevelopmental psychiatric disorders such as schizophrenia (Kirov et al., 2012; Fromer et al., 2014; Purcell et al., 2014). Arc chromosomal microdeletion and intragenic polymorphisms have been found in these disorders, and reduced expression of Arc mRNA has been detected in the frontal cortex of schizophrenia patients (Guillozet-Bongaarts et al., 2014; Hu et al., 2015; Huentelman et al., 2015). These studies suggest the involvement of the Arc pathway in processes associated with psychiatric disorders.

Examining Arc activation dynamics in brain regions vital to behavior will provide the important context for its role in brain function and disease. The frontal cortex plays a crucial role in behavioral control, and its dysfunction is widely implicated in neuropsychiatric disorders (Fuster, 2001; Robbins and Arnsten, 2009; Insel, 2010). Although the mouse frontal cortex is simpler than that of the primate, it contains evolutionarily conserved circuit architecture for top-down control of somatomotor and visceromotor functions, including convergent multisensory inputs, recurrent local connections and strong dopaminergic (DA) neuromodulation (Wise, 2008; Van De Werd et al., 2010; Oh et al., 2014). However, how the neuronal ensembles in the mouse frontal cortex are selected and consolidated for specific information processing tasks remains an unresolved issue. In this article, we provide our perspective through a series of recent studies on frontal cortical circuits which used Arc as a cellular marker to track active neuronal ensembles, and as a molecular probe to modulate neuronal function and behavior.

## Arc Regulates the Emergence of Persistent Firing Patterns in Frontal Cortical Neurons

Motor skill learning engages coordinated activity of neuronal ensembles in the frontal cortex. Different motor behaviors recruit distinct active ensembles, and these ensembles become consolidated with repeated motor learning (Nicolelis and Lebedev, 2009; Dayan and Cohen, 2011; Shmuelof and Krakauer, 2011). During this consolidation process, task-related activities in neurons are either retained or dismissed, in correlation with the animal’s acquisition of motor skills (Costa et al., 2004; Huber et al., 2012; Peters et al., 2014). However, little is known about the molecular and cellular mechanisms by which those learning-related firing changes are regulated.

**Figure 1:**
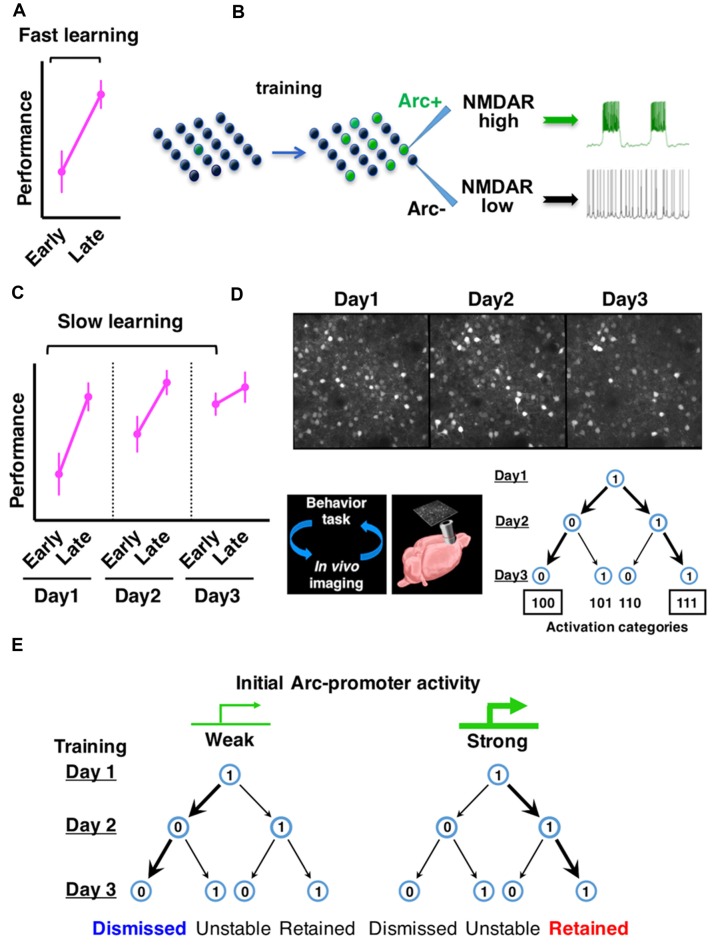
**Arc expression identifies neurons with persistent firing properties and predicts the consolidation of neuronal ensembles in frontal cortex during learning. (A)** Fast motor learning occurs between early and late trials (separated by approximately 1 h) of a training session in the rotarod task. **(B)** Arc expression is induced by motor training in a subset of frontal cortical neurons and NMDA-type glutamate receptor (NMDAR)-mediated persistent firing patterns emerge in this neuronal ensemble. **(C)** Slow motor learning is indicated by performance improvement between early trials conducted on multiple days. **(D)** Repeated *in vivo* imaging of frontal cortex during multiday rotarod training reveals the consolidation of Arc-expressing neuronal ensembles. **(E)** Neurons with relatively weak initial Arc activation are more likely to be dismissed from the ensemble, whereas neurons with relatively strong initial Arc activation are more likely to be retained (Figures are modified with permission from Ren et al., 2014; Cao et al., 2015).

Using a rotarod motor learning task in mice, we examined the effects of prior training on Arc expression and neuronal firing properties (Ren et al., 2014; Figures 1A,B). By imaging an Arc-promoter-driven GFP reporter, we found that motor training induces Arc expression in about 1/3 of excitatory neurons, which are defined by the expression of cortical glutamatergic excitatory neuron marker calcium/calmodulin-dependent protein kinase II alpha (CaMKIIα; Vazdarjanova et al., 2006). We then used fluorescence-guided patch-clamp recording to measure the firing properties of these neurons in *ex vivo* frontal cortical slices. To mimic the physiological environment conducive for neuronal firing in intact frontal cortex, a low concentration of agonists for the NMDA-type glutamate receptor (NMDAR) and dopamine-D1-type receptor (D1R) were included in the slice medium (Seamans and Yang, 2004; Tseng and O’Donnell, 2005; Stewart and Plenz, 2006; Durstewitz and Gabriel, 2007). We found that Arc-GFP+ neurons from motor-trained mice exhibit sustained depolarization and persistent firing, in striking contrast to the Arc-GFP- neurons from the same mice or neurons from the untrained mice (Ren et al., 2014). These results demonstrate that motor training induces Arc expression in a subset of frontal neurons, and that persistent firing patterns emerge in this neuronal ensemble.

What is Arc’s role in persistent neuronal firing? In mice carrying a genetic deletion of the Arc protein, frontal cortical neurons show training-dependent activation of Arc-promoter, but the level of persistent firing in these neurons is greatly diminished, suggesting that the induction of training-related persistent firing depends on Arc. The emergence of persistent firing in Arc-expressing neurons requires NMDAR activity and is associated with Arc-dependent enhancement of NMDAR function, but not changes in intrinsic membrane excitability or AMPA-type glutamate receptor (AMPAR) function (Ren et al., 2014). These findings therefore reveal a new role of Arc distinct from its previously known effect on AMPAR trafficking (Rial Verde et al., 2006; Shepherd et al., 2006; McCurry et al., 2010; Jakkamsetti et al., 2013). The frequency of NMDAR-dependent miniature excitatory postsynaptic currents and the amplitude of NMDA-evoked currents are increased in Arc-expressing frontal cortical neurons (Ren et al., 2014), suggesting a greater number of synapses enriched for NMDARs in these neurons. A recent biochemical study has reported that the crystal structures of Arc subdomains are similar to retroviral Gag protein and provided the molecular basis of Arc binding to NMDARs, including NR2A and NR2B, as well as several other postsynaptic proteins (Zhang et al., 2015). The mechanistic details underlying Arc-dependent regulation of NMDA receptors will be an interesting topic for future studies. Together, current findings suggest an Arc-dependent molecular pathway by which motor training promotes the emergence of NMDAR-mediated persistent firing patterns in specific frontal neuronal ensembles.

## Arc Expression Predicts the Consolidation of Neuronal Ensembles during Motor Learning

At the behavioral level, motor learning typically occurs in two stages (Figures 1A,C). Fast motor learning in the accelerating rotarod task is indicated by enhanced performance in late trials compared to early trials (separated by approximately an hour) on a single day; slow motor learning is characterized by improved performance in early trials conducted over several days (Buitrago et al., 2004; Farr et al., 2006; Rothwell et al., 2014). In Arc knockout mice, although fast motor learning still occurs, slow motor learning is disrupted (Ren et al., 2014; Cao et al., 2015). These results led us to examine further the expression patterns of Arc over the time course of slow motor learning.

By using *in vivo* two photon imaging of Arc-GFP mice (Cao et al., 2013), we tracked the expression of Arc in the same sets of neurons in the same frontal cortical region (M2) over multiple days of rotarod training (Cao et al., 2015). Arc expression is repeatedly induced by daily training session and decays back to the baseline level within a day. The activation patterns of individual neurons over 3 days of rotarod training can be represented by a 3-digit binary string with a “1” (activated) or “0” (not activated) on each day (Figure 1D). Compared to a random daily activation model, there is a selective increase of neurons in the “111” and “100” categories, at the expense of “101” and “110” categories. Consequently, the neurons activated by the initial rotarod training are predominantly consolidated into a persistently retained ensemble (“111”) in motor learning.

Are Arc-expressing neurons consolidated in a task-specific manner? By comparing the rotarod learning task with a free wheel-running task, it was found that Arc activation patterns under the same motor task are much more predictable than that between the different motor tasks, suggesting task-specific activation of Arc ensembles (Cao et al., 2015). During rotarod training, the day-2 Arc activation pattern is more effective than that on day-1 in predicting the reactivation pattern on day 3. This increased predictive effect is specific for rotarod learning, but not seen under free wheel-running conditions or in the home cage, suggesting that Arc-expressing neuronal ensembles are specifically consolidated during motor learning. In Arc knockout mice, the predictive effect of Arc-promoter activation does not increase on the second day of rotarod training compared to the first day, suggesting that the consolidation of neuronal ensembles is impaired in the absence of Arc function.

Moreover, during ensemble consolidation, the initial intensity of Arc expression predicts a neuron’s probability of being retained or dismissed (Cao et al., 2015). Neurons with initially weak Arc activation are more likely dismissed from the ensemble. In contrast, neurons with initially strong Arc activation are more likely retained (Figure 1E). Thus, these studies identify Arc as a key gene that underscores functional ensembles activated in the frontal cortex and predicts cellular participation in the ensemble consolidation process over the course of motor learning (Ren et al., 2014; Cao et al., 2015). Supporting the importance of Arc ensembles in motor learning, a recent study has reported that by disrupting the synaptic connections of Arc-expressing neurons in the frontal cortex, newly acquired motor skills can be selectively erased (Hayashi-Takagi et al., 2015). Arc appears to provide a genetic foothold in frontal cortical neurons, and facilitates functional prediction and mechanistic dissection of neuronal ensembles.

## Dopamine Is Required for the Amplification of Activity-Dependent Arc Expression in Development

**Figure 2:**
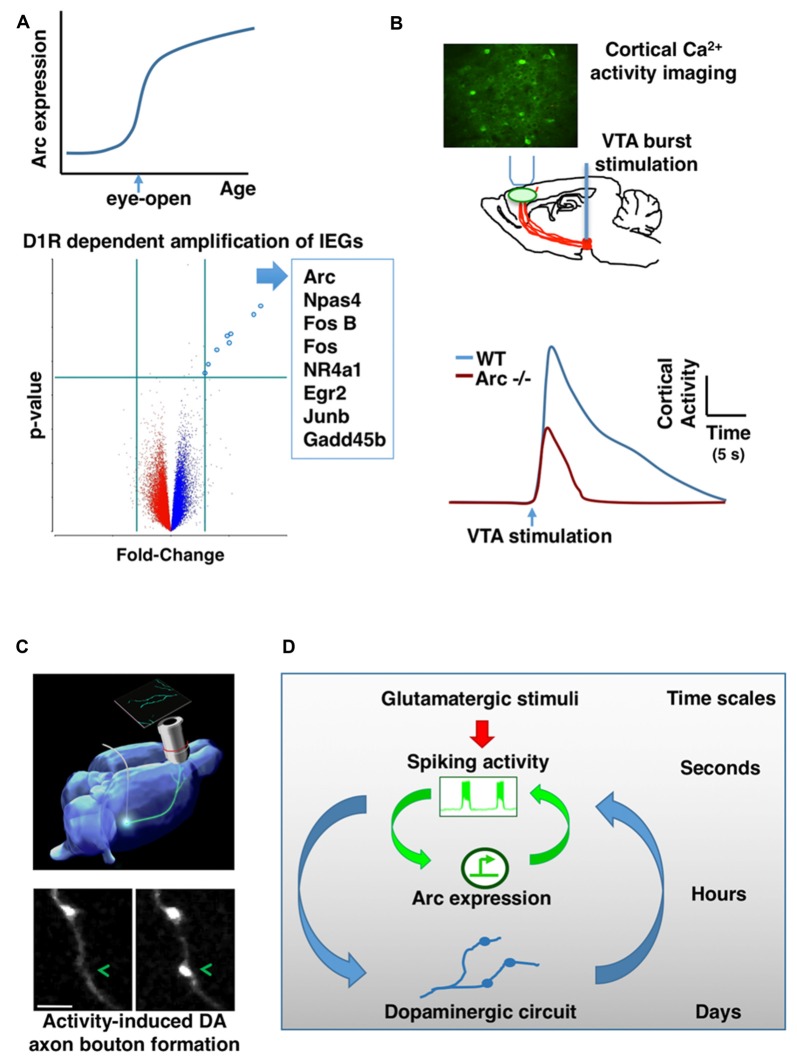
**Mutual interaction between activity-dependent gene expression and dopaminergic (DA) circuit in the frontal cortex. (A)** Arc gene expression in the frontal cortex is sharply amplified when mouse pups open their eyes for the first time during early postnatal development (top). DA input through D1-type receptors (D1R) is required for the activity-dependent amplification of Arc and a list of other IEGs (bottom). **(B)** *In vivo* imaging of genetically encoded calcium indicators in the frontal cortex revealed that ventral tegmentum area (VTA) stimulation-evoked activity is significantly reduced in Arc knockout mice (Arc−/−) in comparison to wild-type mice. **(C)** Phasic activation of dopamine neurons in adolescence promotes the formation of mesofrontal DA axonal boutons. **(D)** A conceptual model showing the hierarchical nesting of regulatory dynamics across multiple time scales and biological levels. Mathematically, the coupling between fast and slow dynamics can be represented by pairs of differential equations: (i) *dx/dt = f(x,y)*; (ii) *dy/dt = μg(x,y)* where *x* describes the state of the fast subsystem, *y* describes the state of the slow subsystem, *f* and *g* are functions of the subsystems, and *μ* ≪ 1 is the ratio of time scales (Izhikevich, 2007). The slower processes in the hierarchy integrate the faster dynamics and reach threshold for activation gradually. But once activated, the effects of slower processes persist longer and modulate the amplitude of the faster dynamics (Figures are modified with permission from Mastwal et al., 2014; Managò et al., 2016; Ye et al., 2016).

The studies discussed above examined the induction and function of Arc over several days of rotarod learning in adult mice. During the postnatal development of mice, other salient behavioral events may occur. How does Arc expression in frontal cortex change during normal development? Using quantitative RNA analysis to map the developmental profile of Arc expression in mice, we found that when mouse pups open their eyes for the first time near the end of the second postnatal week, Arc mRNA levels in the frontal cortex rise sharply (Ye et al., 2016; Figure 2A). As we suspected, visual stimuli are required to induce the amplification of Arc mRNA at this age. Less expected is our finding that, before eye-opening, electrical neural stimulation alone—applied to see whether lack of neuronal stimulation might account for the absence of Arc mRNA at this age—is insufficient to amplify Arc mRNA in the frontal cortex.

Historically, Arc gene was first cloned from adult brain tissue according to its strong induction in response to electrical seizure activity (Link et al., 1995; Lyford et al., 1995). In addition to electrical activity, Arc expression in adult brain is also affected by several neuromodulators in a regional specific manner (Fosnaugh et al., 1995; Pei et al., 2000; Sanders et al., 2008; Soulé et al., 2012). The frontal cortex receives extensive DA innervation (Verney et al., 1982; Kalsbeek et al., 1988) and activation of dopamine D1-type receptors (D1R) can enhance *Arc* transcription in adult brain (Fosnaugh et al., 1995). However, some *in vitro* studies have suggested that dopamine’s role in Arc induction is not essential. In cultured cells derived from embryonic hippocampal or cortical tissues, electrical activation alone appears to be sufficient to trigger Arc expression through calcium influx from NMDARs or voltage-gated calcium channels (Shepherd et al., 2006; Bloomer et al., 2008). Nevertheless, considering the anatomical and developmental origins of these cells and their culture status, it is difficult to predict whether DA signaling would play any role in the amplification of Arc mRNA during early postnatal development of the frontal cortex.

DA axons are already present in the frontal cortex in the first postnatal week (Verney et al., 1982; Kalsbeek et al., 1988) and their density increases significantly during subsequent postnatal development (Ye et al., 2016). Our recent *in vivo* studies demonstrate that DA signaling is required for the amplification of Arc mRNA in response to neural activity during frontal cortical development (Ye et al., 2016). Neurochemical lesion of dopamine neurons in the ventral tegmentum area (VTA) prevents the amplification of Arc expression during postnatal development of the frontal cortex; D1R antagonist acutely inhibits the amplification of Arc mRNA by visual experience at the time of eye-opening on P13; and before eye-opening, D1R activation is required to enable the amplification of Arc expression in response to electrical stimuli. Not only for Arc, D1R signaling is also required for the induction of a list of other IEGs in early postnatal frontal cortex (Figure 2A). Dopamine may affect several steps in the pathways leading to activity-dependent gene expression, including neuronal excitability and intracellular signaling (Seamans and Yang, 2004; Tritsch and Sabatini, 2012). Particularly, D1R activation is known to engage cAMP and PKA signal transduction pathway, and activation of this pathway can enhance the level of Arc mRNA in neuronal cultures (Waltereit et al., 2001). Together, these findings reveal an important role of dopamine in the amplification of activity-dependent Arc expression, and suggest that synergistic electrical and DA activity is crucial for the establishment of normal activity-dependent gene expression pattern during frontal cortical development.

## Genetic Disruption of Arc Causes Alterations in the Dopamine System and Neuropsychiatric Deficits

Perturbations of DA signaling have been associated with the pathogenesis or treatment of neurodevelopmental psychiatric disorders (Tritsch and Sabatini, 2012; Slifstein et al., 2015). In parallel, Arc signaling complex has been shown as a target of mutations in neurodevelopmental psychiatric disorders such as schizophrenia (Fromer et al., 2014; Purcell et al., 2014; Hu et al., 2015; Huentelman et al., 2015). Our findings on the relationship between dopamine and Arc at an early life stage provide a novel intersection point between two disease-associated molecular pathways (Ye et al., 2016). But what is the functional significance of Arc in schizophrenia-related neurobehavioral phenotypes and brain circuits?

In collaboration with Drs. Francesco Papaleo and Daniel Weinberger, we have conducted a broad range of behavioral tests in Arc knockout mice (Managò et al., 2016). While Arc genetic disruption does not affect general health and lower-level reflexive behaviors, it causes a number of higher level behavioral deficits consistent with schizophrenia-related phenotypes, including deficits in sensorimotor gating, social behaviors, cognitive functions, and amphetamine-induced psychomotor responses. Aspects of these behaviors have been suggested under DA regulation (Arguello and Gogos, 2006), raising the possibility that the DA pathway might be impaired in Arc knockout mice.

The mesofrontal DA circuit is involved in the control of motivated behaviors and cognitive functions (Björklund and Dunnett, 2007; Robbins and Arnsten, 2009; Luna et al., 2015). Abnormal development of this pathway has been proposed to contribute to several neurodevelopmental psychiatric disorders (Chambers et al., 2003; Winterer and Weinberger, 2004; Casey et al., 2010). Particularly, human molecular imaging studies have indicated a hypoactive frontal DA system in schizophrenia patients (Howes and Kapur, 2009; Slifstein et al., 2015).

In addition to schizophrenia-related behavioral phenotypes, we found that genetic disruption of Arc in mice reduces frontal dopamine release (Managò et al., 2016). In further support of this finding, two-photon imaging of genetically encoded calcium indicators in the frontal cortex revealed that VTA stimulation-evoked activity is significantly reduced in Arc mutant mice in comparison to wild-type mice (Figure 2B). The normal response in wild-type mice depends on D1R activation; and application of a D1R agonist in Arc mutant mice alleviates their response deficiency. Together, these results suggest a hypo-dopamine state in the frontal cortex of Arc mutant mice, consistent with human imaging studies of schizophrenia patients (Slifstein et al., 2015). Both the mesofrontal circuit deficits and the cognitive dysfunctions in Arc knockout mice were rescued by D1R agonist in the frontal cortex, suggesting a role for Arc in regulating normal frontal DA neurotransmission and related cognitive behaviors. Certain populations of excitatory neurons in the frontal cortex project back to VTA dopamine neurons (Carr and Sesack, 2000; Watabe-Uchida et al., 2012; Beier et al., 2015). As Arc is abundantly expressed in cortical excitatory neurons but not detected in midbrain dopamine neurons (Shepherd and Bear, 2011), Arc might play a role in regulating activity-dependent maturation of the mesofrontal circuits during postnatal development.

## Activity-Dependent Plasticity of Mesofrontal DA Circuit during Adolescence

Midbrain DA neurons affect numerous brain processes via different projections and firing patterns (Björklund and Dunnett, 2007). For example, neurons in the VTA fire in bursts to reward-associated or motivationally salient stimuli. While VTA DA innervation of the nucleus accumbens reaches maturity relatively early, the projections to the frontal cortex exhibit a more protracted maturation through adolescence (Verney et al., 1982; Kalsbeek et al., 1988; Naneix et al., 2012).

Our recent study has shown that this development is influenced by experience (Mastwal et al., 2014). Particularly, voluntary wheel running behavior, which is known to stimulate phasic firing of VTA DA neurons, promoted the formation of boutons on mesofrontal axons in adolescent, but not adult, mice. Moreover, this effect was mimicked by phasic, but not tonic, optogenetic stimulation of DA neurons in the VTA (Figure 2C). This adolescent axonal plasticity is regulated by both DA and glutamatergic transmission. The enhancement of dopamine bouton formation was further correlated with sustained activation of frontal cortex following VTA stimulation and a corresponding suppression of psychomotor response to amphetamine. Together, these findings indicate that the adolescent mesofrontal circuit is particularly susceptible to phasic activity-induced structural and functional modification. This adolescent plasticity may facilitate the normal experience-dependent strengthening of frontal DA input and behavioral control.

## Conclusion

The series of studies described above suggest several regulatory mechanisms in the emergence and consolidation of frontal cortical neuronal ensembles. First, Arc expression is induced in a behavioral task-specific manner, regulates persistent firing of frontal cortical neurons, and predicts the consolidation of neuronal ensembles during learning. Thus, the Arc pathway presents a prototypical example of an activity-dependent genetic feedback mechanism in the regulation of neuronal ensembles. Second, the developmental emergence of activity-dependent Arc expression depends on DA input, and the normal functioning of frontal dopamine circuits in turn requires the Arc gene. This mutual interaction suggests an auto-regulatory mechanism to amplify the impact of neuromodulators and activity-regulated genes during postnatal development. Third, the maturation of the frontal DA circuit extends through adolescence and is susceptible to activity-dependent modification during this period. This developmental plasticity may help explain the circuit deficits caused by genetic disruption of Arc pathway, and suggest activity-guided therapeutic strategies to ameliorate such deficits.

While this perspective article has focused on the role of Arc in glutamatergic and DA signaling in frontal cortex as a well-characterized model for activity-dependent genetic feedback, recent studies have also provided exciting insights into the functions of other IEGs in both excitatory and inhibitory neuronal circuits (Leslie and Nedivi, 2011; Sun and Lin, 2016). Particularly, it has been reported that neuronal activity induces distinctive gene expression programs in excitatory and inhibitory neurons (Spiegel et al., 2014; Mardinly et al., 2016). While some of the early response genes, such as transcription factors Npas4 and c-Fos, are similarly induced in both excitatory and inhibitory neurons, late response genes show more divergence between cell types. Through these late response genes, such as secreted molecules PTX2 and BDNF (Huang et al., 1999; Chang et al., 2010), activity-induced Npas4 can regulate synaptic input to both types of neurons to maintain circuit wide homeostasis (Spiegel et al., 2014).

Taken together, the basic logic governing the time evolution of neuronal ensembles appears to feature a hierarchical nesting of regulatory dynamics across multiple time scales and biological levels, from fast fluctuations in neuronal firing and neuromodulatory signaling to slow gene expression and circuit connectivity changes (Figure 2D). The slower processes in the hierarchy integrate the faster dynamics and reach threshold for activation gradually. But once activated, the effects of slower processes persist longer and modulate the amplitude of the faster dynamics. Such an interlinked construction of fast and slow feedback loops can enable rapid response to environmental stimuli and robust representation of experiential regularity (Brandman et al., 2005; Izhikevich, 2007; Kiebel et al., 2008). In addition, it appears that developmental stage will be an important factor regulating the relative contributions of these processes. Future studies to further elucidate the mechanistic details and constraints in this model may help our understanding of the intermediate circuit phenotypes linking psychiatric risks to brain function, inspire the design of novel neuromodulatory approaches through activity-guided ensemble manipulation, and provide model experimental systems to test translational strategies.

## Author Contributions

KHW, SM and VC wrote the article and generated the figures.

## Funding

This work was supported by the National Institute of Mental Health Division of Intramural Research Programs ZIA MH002897.

## Conflict of Interest Statement

The authors declare that the research was conducted in the absence of any commercial or financial relationships that could be construed as a potential conflict of interest.
